# Dataset on the long-term monitoring of foundation vertical deformations on medium-expansive soil

**DOI:** 10.1016/j.dib.2025.111422

**Published:** 2025-02-21

**Authors:** Hawkar Hashim Ibrahim, Rizgar Ali Hummadi

**Affiliations:** Department of Civil Engineering, College of Engineering, Salahaddin University-Erbil, 44002 Erbil, Kurdistan Region, Iraq

**Keywords:** Expansive clay, Ground movement, Swelling, Swell-shrinkage, Rainfall

## Abstract

This paper presents a complete data set from the long-term field monitoring of vertical deformation in four footings resting on medium-expansive soil. The four key variables (vertical deformation, daily average air temperature, weekly cumulative rainfall, and soil water content at a depth of 60 cm) were recorded over a period of 974 days. The vertical deformations were measured with high-precision dial gauges. At the same time, the advanced instruments, Bosch GLL 3-80 G Professional line laser and LEICA DNA 10 digital levels were cross-used for measurement to ensure the accuracy and reliability of the results. The data collection was designed to include the effects of expansive soil properties, such as swelling during wet seasons and shrinkage during dry seasons. This is necessary for understanding the soil-structure interaction under natural field conditions, which differs considerably from controlled laboratory studies. This dataset presents a great possibility of being reused by researchers to support further studies on soil-structure interaction, develop predictive models for expansive soils, and analyze long-term structural stability. It is particularly useful in developing machine learning algorithms that can be used to predict foundation behavior in response to different environmental conditions, optimize foundation designs on expansive soils, and specifically predict foundation heave. The availability of this dataset provides an invaluable resource in advancing geotechnical engineering research.

Specifications Table

**Table:** 

Subject	Earth and Planetary Sciences
Specific subject area	Geotechnical Engineering and Engineering Geology
Type of data	Table
Data collection	The data was collected for 974 days of the performance of a structure supported by four footings while considering four variables. The first variable is the daily average air temperature, which gives information about the climatic fluctuations and their probable effect on soil behavior, and the second one is the weekly cumulative rainfall. They were obtained from the General Directorate of Agriculture, Erbil, under the Ministry of Agriculture and Water Resources of the Kurdistan Regional Government (KRG). These data are necessary for monitoring the performance of foundations on expansive soils. In addition, the water content at a depth of 60 cm was the third variable that was recorded in order to evaluate the moisture levels in the soil near the foundation footings. This was helpful in understanding the interaction between the variations of soil moisture and the behavior of expansive soil. The fourth variable was vertical deformations, which were measured by dial gauges that monitored the long-term upward and downward movements of the footings. Furthermore, highly accurate advanced instruments, such as the line laser Bosch Professional GLL 3-80 G and LEICA DNA 10 digital levels, have been used to cross-validate these deformation measurements to ensure the accuracy and reliability of these deformation measurements. Detailed descriptions of the equipment and procedures used for these measurements can be found in the Experimental Design, Materials, and Methods section.
Data source location	The vertical deformation measurements and soil water content were carried out inside the College of Engineering at Salahaddin University-Erbil (36°08′37.4"N 44°01′32.7"E). In contrast, the data on weekly accumulated rainfall and daily average air temperature were obtained from the General Directorate of Agriculture in Erbil (36°10′18.4"N 44°00′52.0"E).
Data accessibility	All data are available in a public repository: Repository name: Mendeley Data Data identification number: DOI: 10.17632/2t2xf85rnd.1 Direct URL to data: https://data.mendeley.com/datasets/2t2xf85rnd/1 Instructions for accessing these data: -
Related research article	*None*

## 1 Value of the Data

• Long-term swell-shrinkage studies are difficult to conduct on actual models, as they would be too costly and time-consuming.
• The vertical deformations developed in full-scale foundations may be compared with those of small-scale laboratory samples, and correlations may be drawn.
• This dataset has great potential for researchers to further the work into prediction models with machine learning techniques.
• Long-term field data is very useful in understanding the reasons why some structures on expansive soil have not developed serious structural problems over time.

## 2 Background

Expansive soils are very sensitive to the change in moisture content, which is widely distributed around the world [1,2]. This type of soil swells and shows considerable volumetric deformation in response to changes in moisture content [[3], [4], [5]]. As a result, damage and distortion to structures from the swell of expansive soils due to changes in moisture conditions are common problems that frequently occur in many parts of the world, particularly for light buildings and pavements [[6], [7], [8]]. For this reason, information related to the soil movement over time is of practical interest for the reliable design of foundations for structures on expansive soils. Therefore, many researchers have recently tried to improve expansive soil by using various techniques in the laboratory [[9], [10], [11], [12], [13]]. The prediction of swelling behavior in the laboratory is available in the literature using oedometer equipment. However, this swelling does not represent the behavior of the actual field because the sample is restrained laterally. Therefore, a concrete frame supported by four real foundations was built on medium-expansive clay to monitor the vertical deformations of foundations due to variations in rainfall. The total period of monitoring was 974 days. The concrete frame was designed in such a way that it faced variable weather conditions during the period of the study.

## 3 Data Description

The dataset (Excel Worksheet.xlsx) contains four sheets: daily average air temperature, weekly cumulative rainfall, soil water content (SWC), and vertical deformations. It was collected from November 2021 to July 2024 and gives an extended view of the atmospheric and subsurface conditions in the study region. Each variable is described in detail below.

### 3.1 Daily average air temperature

This sheet contains information on the record of temperature measured per day in degrees Celsius. The data is systematically and chronologically organized for a long observation period, containing date, number of days, and daily average air temperature (°C) fields to ensure clarity and ease of analysis. The temperature data serves as an important basis for assessing temporal climatic changes; therefore, a possible relation between temperature fluctuations, soil behavior, and the resulting vertical deformation of foundation structures can be established. In addition, this data was obtained from the General Directorate of Agriculture in Erbil, operating under the Ministry of Agriculture and Water Resources, KRG.

### 3.2 Weekly cumulative rainfall

This is a record of rainfall in millimeter measures, aggregated on a week-to-week basis. This data was obtained from the General Directorate of Agriculture in Erbil and then arranged based on weekly cumulative rainfall. It is presented in this manner to be clear and enable the analyst to conduct a longitudinal analysis of the same, if needed, with fields like Date, Day, and Rainfall (mm). Such documentation of precipitation over time allows this dataset to comprehensively portray the seasonal rainfall trend and its variability across the monitoring period. This is particularly valuable information for understanding how rainfall influences soil moisture dynamics, which in turn affects the stability and behavior of foundation structures.

Rainfall is one of the most important inputs for studying the climatic event-geotechnical response interactions, such as soil swelling and shrinkage variations. Besides that, it enables the determination of periods of high precipitation that may coincide with significant vertical deformations in the foundation footings, providing some indications of cause-and-effect relationships. It supports the analysis of seasonal impacts on soil behavior and is also useful in long-term performance predictions of structural systems under time-varying environmental conditions.

### 3.3 Water content at a depth of 60 cm

The dataset is a detailed record of measurements important in developing fundamental theories relating environmental conditions to foundation performances. The Soil Water Content (SWC) 60 cm depth dataset has measurements recording the soil moisture percentages accurately taken from several footing locations referred to as Footing_1, Footing_2, Footing_3, and Footing_4. This data is tabulated in Date, Day, and SWC values, forming the basis for the interaction between moisture content variation and expansive soil behavior.

Soil moisture content is one of the most important parameters in geotechnical studies involving expansive soils, which develop significant volumetric changes under varying soil moisture conditions. In this light, this dataset provides insights into how SWC temporally changes and enables investigation of the effect of rainfall amount and other climatic conditions on soil–water interactions. It does this by updating changes that are occurring within soil moisture due to swelling and shrinking in relation to the possible effect directly on the foundations through disturbance of stability or loss of integrity. Besides that, this dataset helps conduct the spatial analysis by comparing levels from one footing location against the others, depicting all variabilities usually brought forth by a possibility of differential settlement or any probable structural deformations. This information is important not only for diagnosing the current behavior of the soil but also for developing predictive models and strategies to mitigate the potential risks associated with expansive soil movements; such insights are necessary to design resilient foundations and long-term structural stability under dynamic environmental conditions.

### 3.4 Vertical deformation

This dataset has been recorded over a monitoring period of 974 days using a careful measurement technique to have exact structural variations in the structure over time. The main instrument used was the dial gauge (with an accuracy of 0.01 mm); this is a reliable instrument used to measure changes in displacement. These were further crosschecked using more advanced instruments such as the line laser GLL 3-80 Professional, known for its great precision in leveling and alignment, and the LEICA DNA 10 digital levels to obtain very accurate height measurements. The validation by multiple instruments ensures the quality of this dataset through the reduction of measurement errors and the output of consistent and repeatable results throughout the observation points.

## 4 Experimental Design, Materials and Methods

### 4.1 Study area

**Fig. 1:**
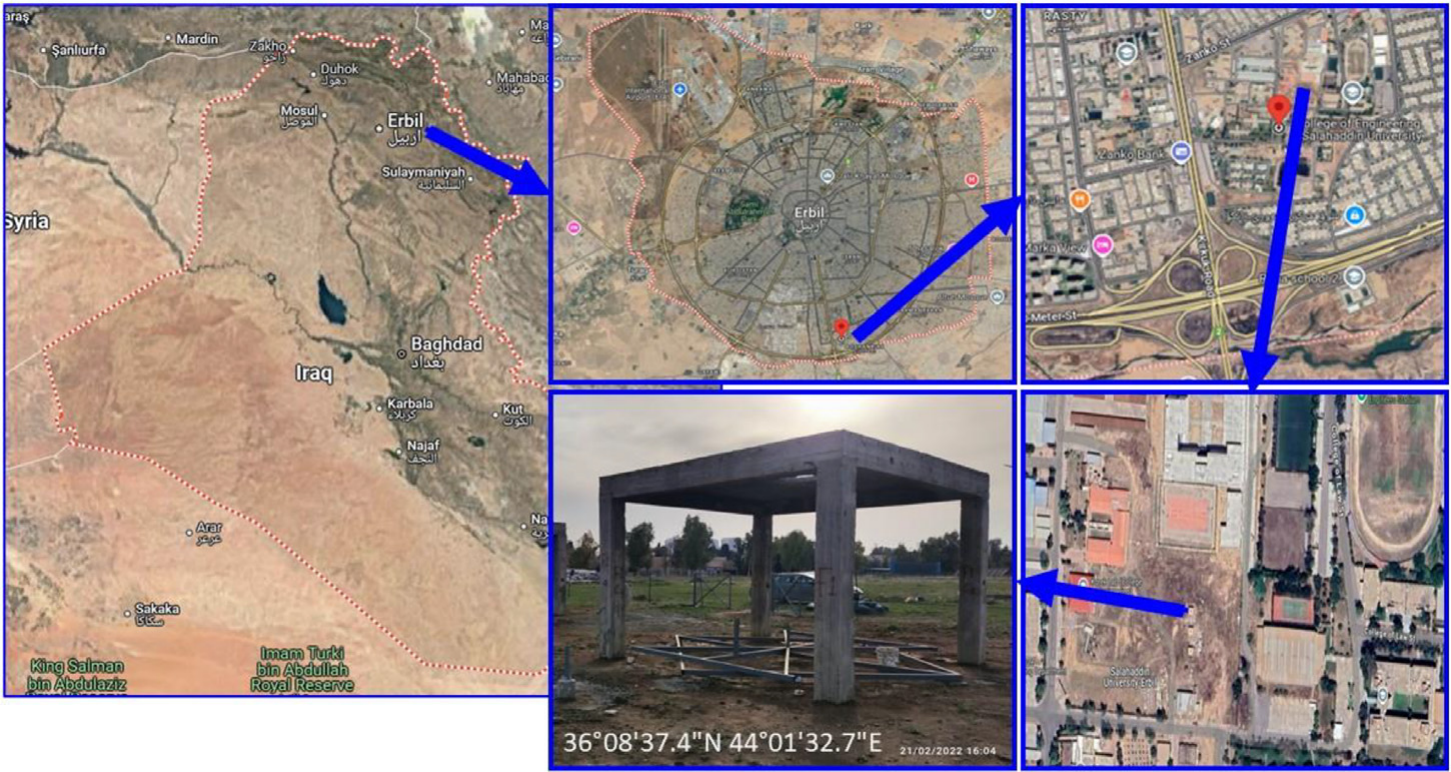
Geographical location and site details of the concrete frame in the College of Engineering at Salahaddin University -Erbil (36°08′37.4"N 44°01′32.7"E), in Erbil city, Iraq.

The study was carried out on a concrete frame structure supported on four single footings within the College of Engineering, Salahaddin University-Erbil, Kurdistan Region, Iraq, at geographical coordinates 36°08′37.4"N 44°01′32.7"E (as shown in Fig. 1). This site, in the southern part of Erbil city, was chosen based on its soil characteristics, medium-expansive soil, and proximity to controlled research facilities. In addition, based on the laboratory tests conducted, the geotechnical properties of the medium-expansive soil sample were determined (see Table 1), and the values presented in the table indicate that the soil can be described as medium-expansive. Such soils are sensitive to moisture fluctuations and undergo substantial volume changes due to swelling during wet conditions and shrinkage during dry periods.

**Table 1:** Geotechnical properties of the medium-expansive soil sample.

Property	Quantity	Standard designation
Specific Gravity	2.69	ASTM_D854 [14]
Passing sieve No. 200, [%]	92	ASTM_D6913 [15]
Gravel, [%]	0	ASTM_D6913 [15]
Sand, [%]	8	ASTM_D6913 [15]
Silt, [%]	72.5	ASTM_D7928 [16]
Clay, [%]	19.5	ASTM_D7928 [16]
Liquid Limit, LL [%]	44	ASTM_D4318 [17]
Plastic Limit, PL [%]	23	ASTM_D4318 [17]
Plasticity Index, PI [%]	21	ASTM_D4318 [17]
USCS Classification	CL	ASTM_D2487 [18]
Compression Index (C_C_)	0.188	ASTM_D2435 [19]
Swelling Index (C_s_)	0.016	ASTM_D4546 [20]

**Fig. 2:**
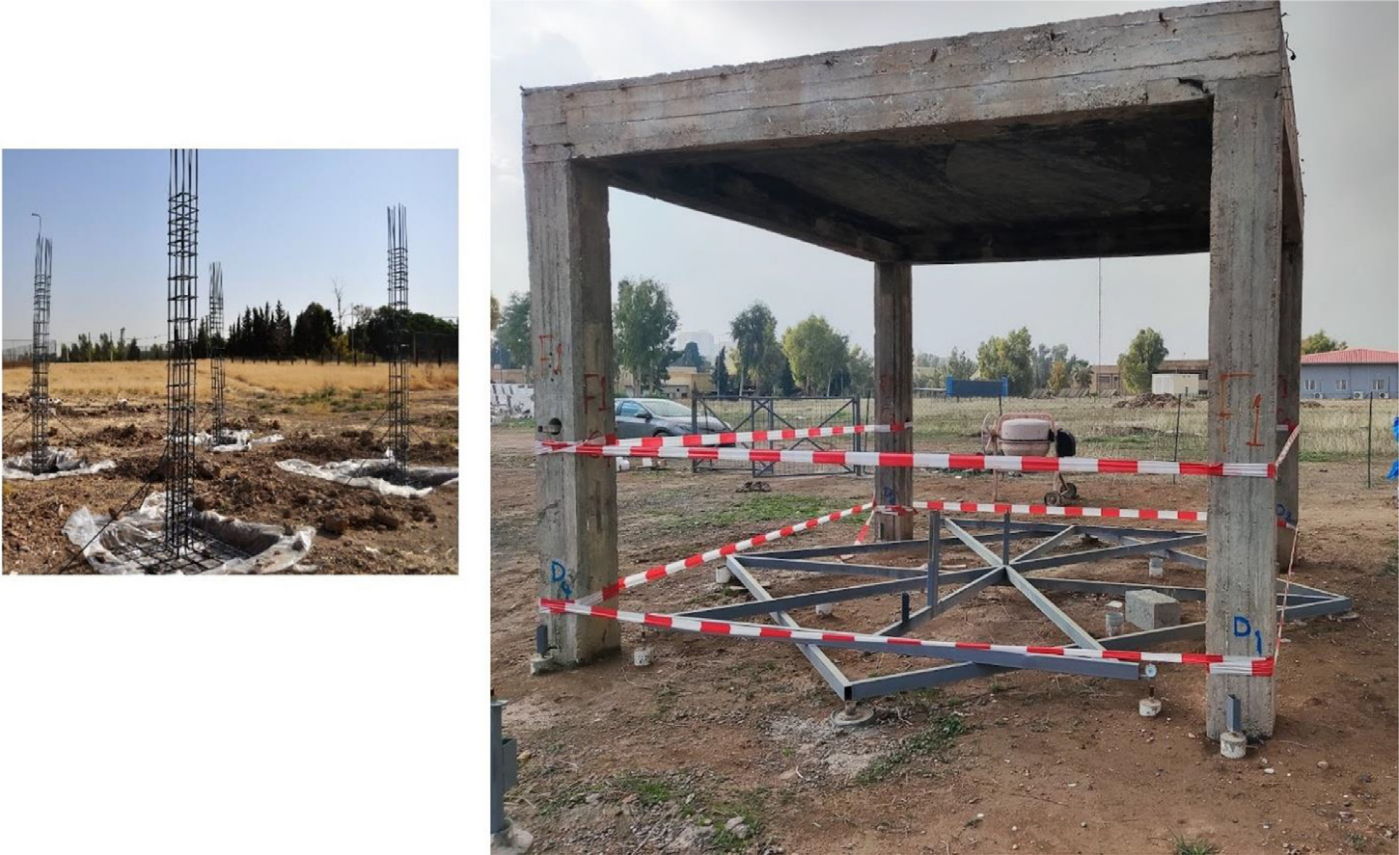
Reinforced concrete frame with four isolated footings for long-term vertical deformations monitoring.

The concrete frame structure was field fabricated with a square base of 430 cm × 430 cm and a total height of 342 cm. The framework consists of four uniformly spaced vertical columns that are connected at the top by horizontal beams, making sure the load is properly transferred for structural stability. Each column rests on one isolated footing of 125 cm by 125 cm to transmit the loads effectively to the foundation soil (see Fig. 2). The frame is also suitable for investigating the fundamental vertical movement of foundations using medium-expansive soils under different environmental conditions. Furthermore, each footing is isolated so that deformation due to medium-expansive soil can be independently monitored.

### 4.2 Field preparation

**Fig. 3:**
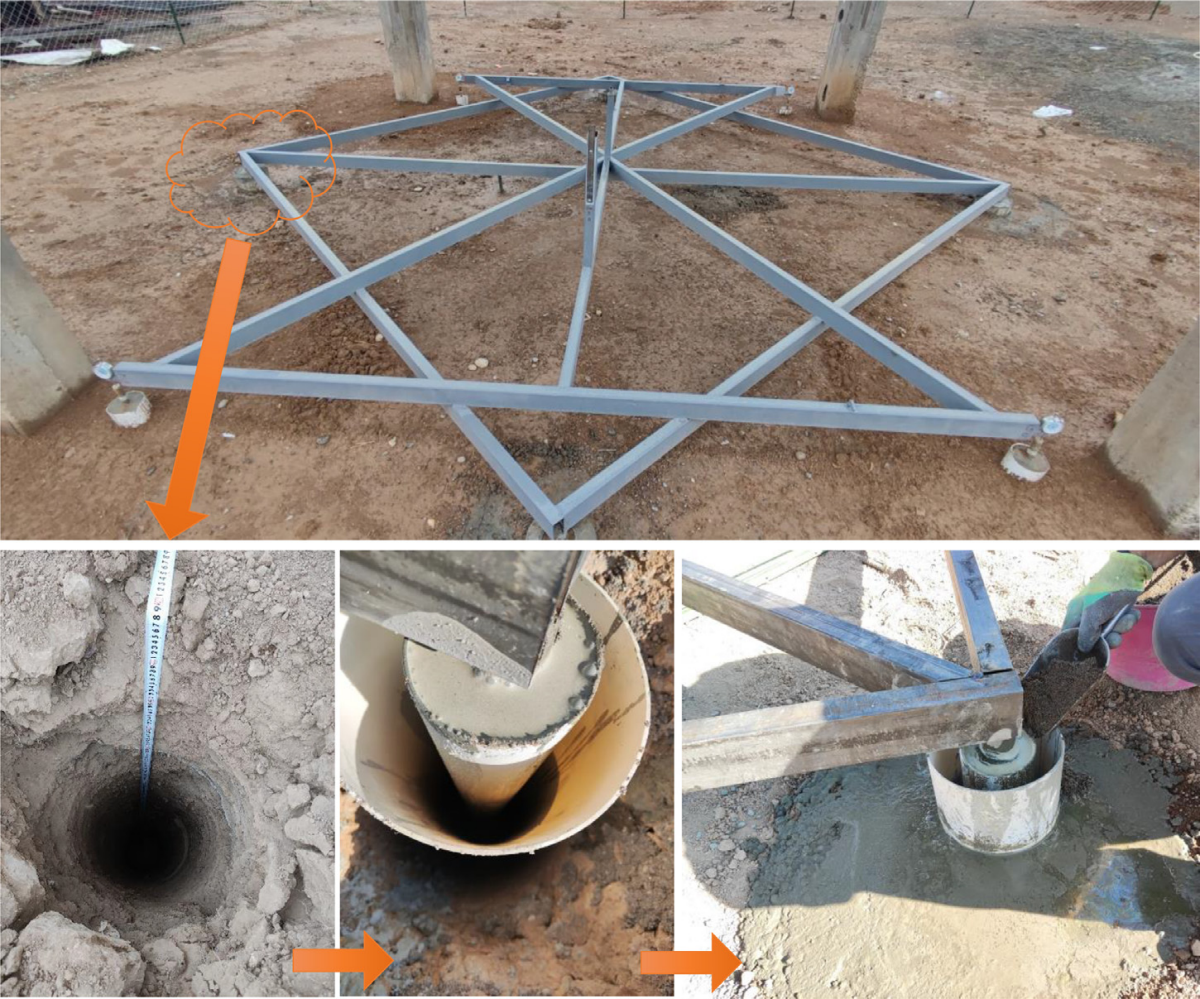
A steel frame system was installed to monitor the foundations of the concrete frame, supported by four steel supports extending down to approximately 300 cm.

**Fig. 4:**
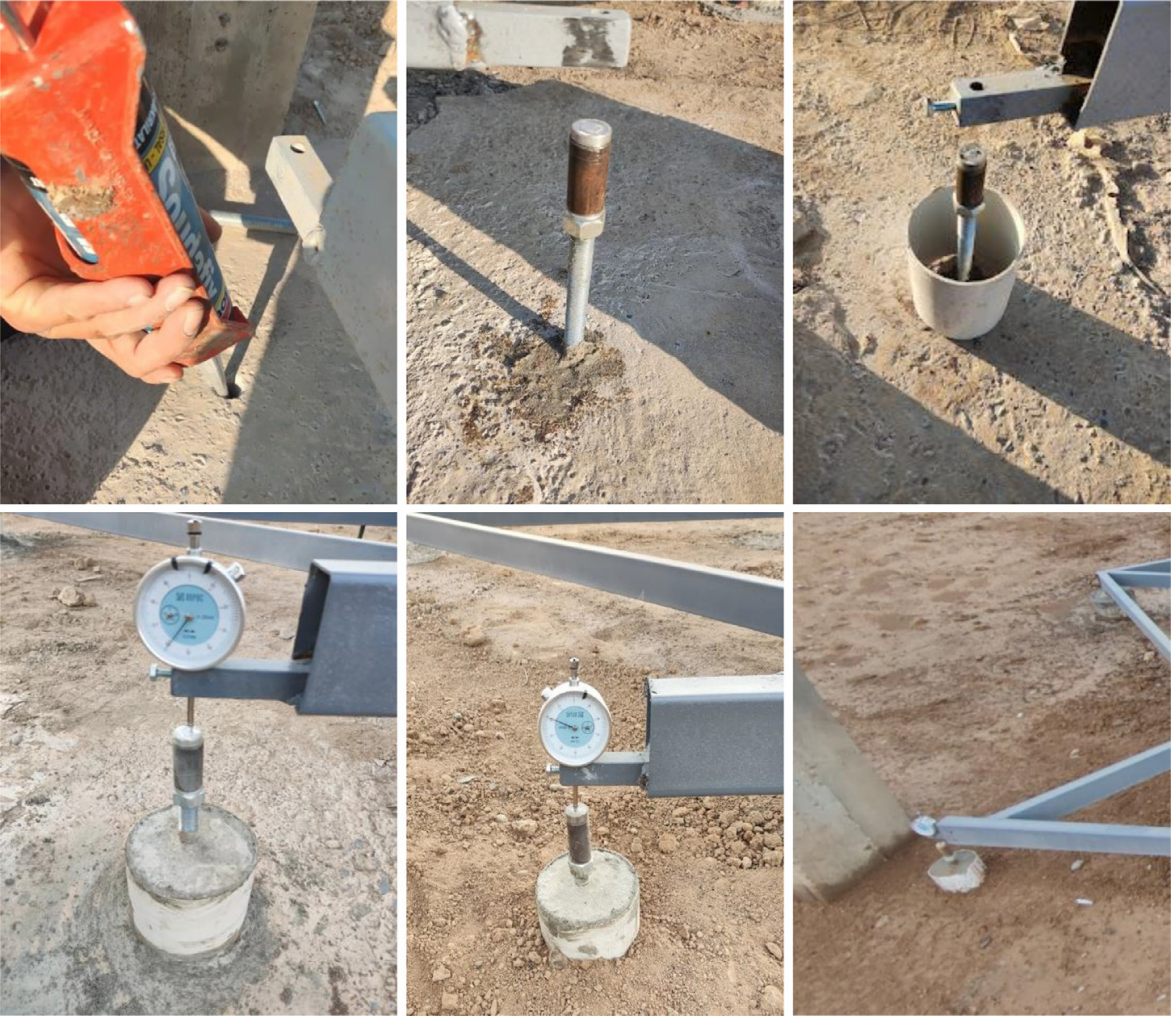
A dial gauge was mounted with the steel frame on each of the four footings for vertical deformation measurement.

A steel frame system was installed to monitor the foundations of the concrete frame, which was supported by four steel supports extending to a depth of approximately 300 cm. This configuration was designed in such a way that the soil swelling effect would be negligible on the measurements recorded by the dial gauges, which were used to measure vertical deformations, as shown in Fig. 3. A dial gauge with a high precision of 0.01 mm was mounted on each of the four footings to measure vertical deformations. This setup allowed for continuous and precise monitoring of any upward or downward movement of the footings, as shown in Fig. 4.

**Fig. 5:**
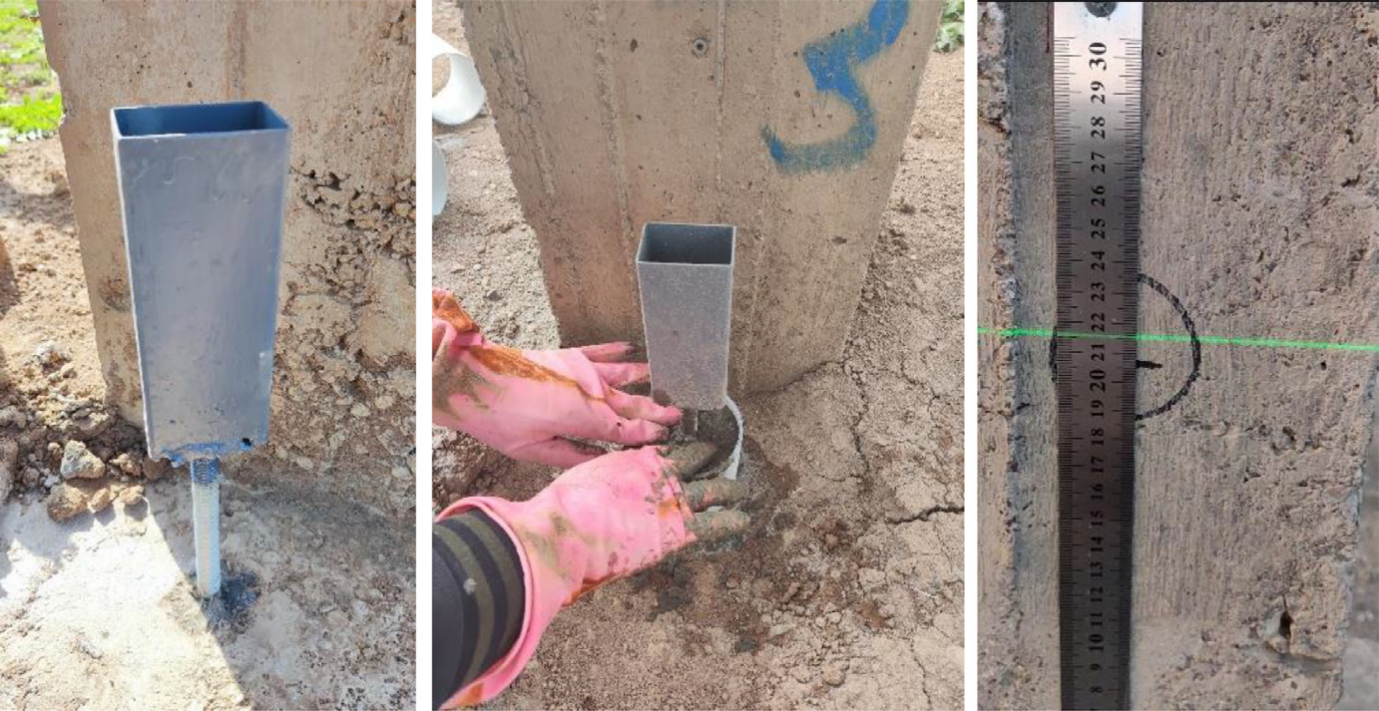
Installation of fixed staff position and steel rule on each column.

**Fig. 6:**
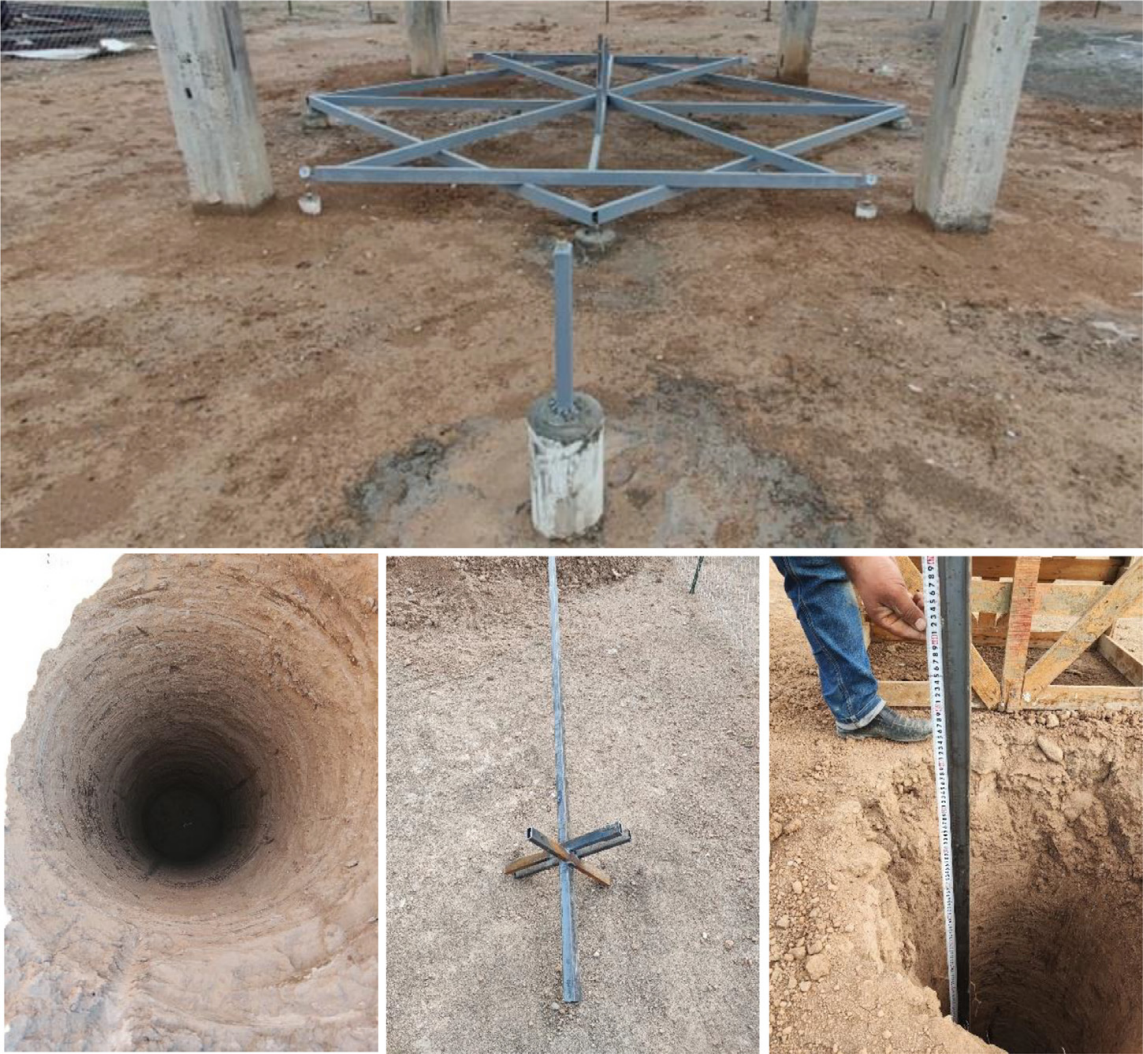
Benchmarks built with deep boreholes and embedded steel pipes for checking vertical deformation measurements.

In addition, each footing was installed on a fixed staff position, serving as a reference for deformation reading. A steel rule was attached to each column to enhance the accuracy in monitoring, as illustrated in Fig. 5, providing the vertical deformation with reference to benchmarks. These benchmarks were prepared with care by digging boreholes vertically from the ground surface with a diameter of 30 cm and extended downward for a depth of 450 cm. Inside each borehole, a square steel pipe was fixed by concrete to provide a rigid and dependable reference point, as indicated in Fig. 6. Thus, this comprehensive setup enabled precise measurement of vertical deformations of the footings with respect to fixed and unmovable benchmarks for long-term monitoring.

### 4.3 Data collection

The data collection process in this study was designed and implemented with great care to ensure that a robust, accurate, and reliable dataset was generated for long-term monitoring of foundation performance. It was mainly intended to measure the vertical deformation of four footings constructed on medium-expansive soil. The focus was the measurement of vertical deformation of four footings constructed on medium-expansive soil and correlating in a consistent way with critical environmental and soil conditions, including cumulative rainfall, daily average air temperature, and soil water content (SWC) at a depth of 60 cm below ground surface. Such parameters were chosen because these would have a highly influencing factor in expansive soils, which swell with wetting and shrink when drying.

These objectives were achieved through the combined use of advanced instruments and validation techniques. High-precision dial gauges were installed on each footing to measure even the smallest vertical displacements, thus ensuring a measurement accuracy of 0.01 mm. These readings were periodically cross-checked with line lasers and digital leveling tools to reduce errors and enhance the datasetʼs reliability. Measurements of SWC were performed to capture the fluctuations in moisture content, which are quite crucial for swelling and shrinkage in soils. Other environmental data, such as rainfall and air temperature, were obtained continuously from reliable government agencies to monitor and understand various factors governing the vertical deformation of the footings.

**Fig. 7:**
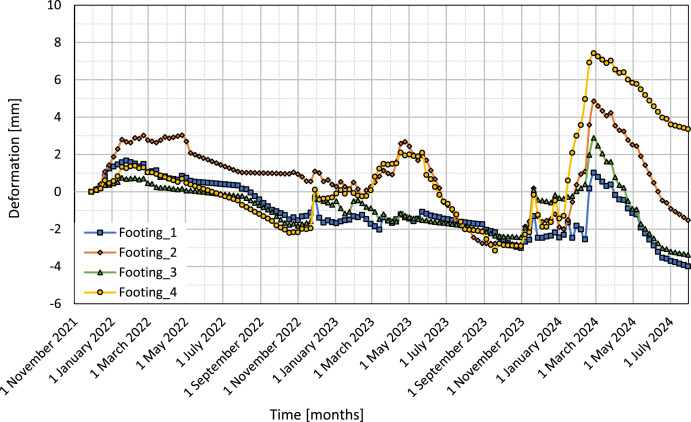
Vertical deformation curves for four footings over time.

Fig. 7 presents the vertical deformations of four footings, Footing_1, Footing_2, Footing_3, and Footing_4, from November 2021 to July 2024. The y-axis is the vertical deformation in mm, and the x-axis is time in months. Positive deformations are for heaving, while negative deformations show settlement.

**Fig. 8:**
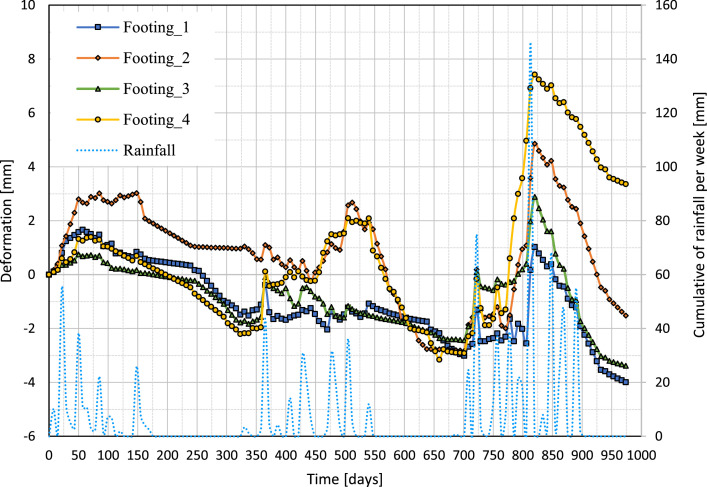
Variations of vertical deformation of the footings versus weekly cumulative rainfall with time.

Fig. 8 represents the relation between cumulative weekly rainfall (right y-axis) and vertical deformations of four footings (left y-axis) within the period of about 1000 days. The dotted blue line is the cumulative weekly rainfall throughout the duration of the study.

**Fig. 9:**
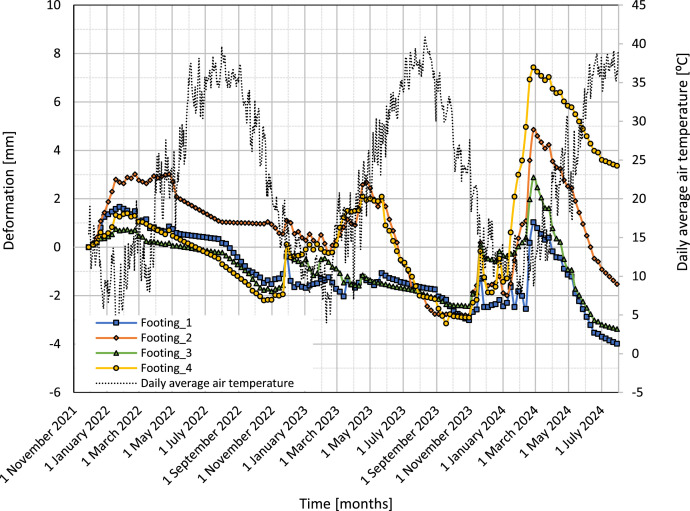
Variation of vertical deformation of the footings with the daily average air temperature and time.

Fig. 9 presents the daily average air temperature, dotted line, right y-axis versus vertical deformation of four foundation footings, and left y-axis for the period of November 2021–July 2024.

**Fig. 10:**
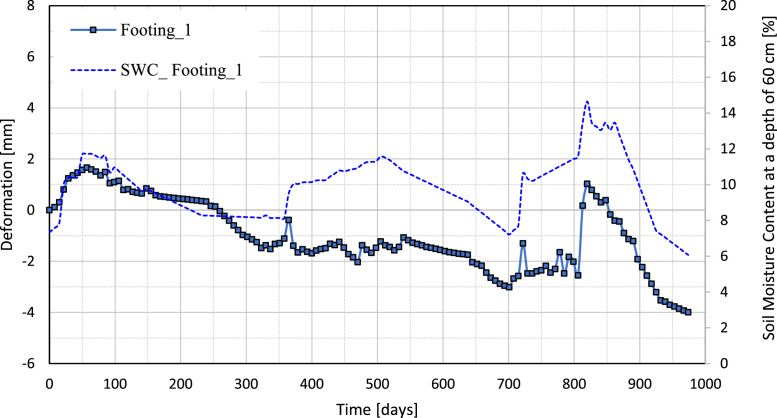
The graph shows the relation between vertical deformation and soil moisture content at a depth of 60 cm for footing_1.

**Fig. 11:**
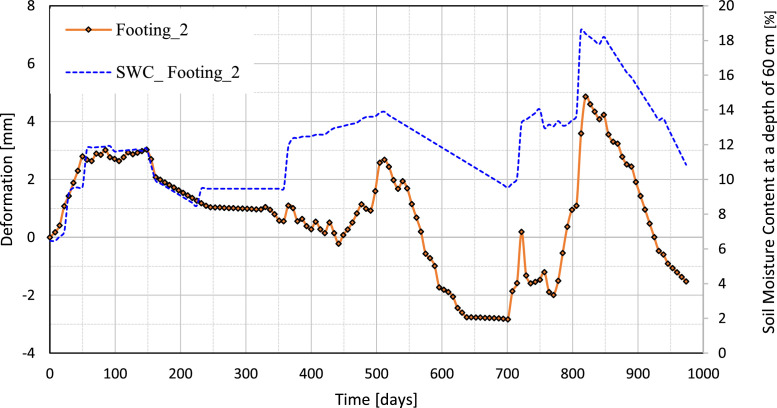
The graph shows the relation between vertical deformation and soil moisture content at a depth of 60 cm for footing_2.

**Fig. 12:**
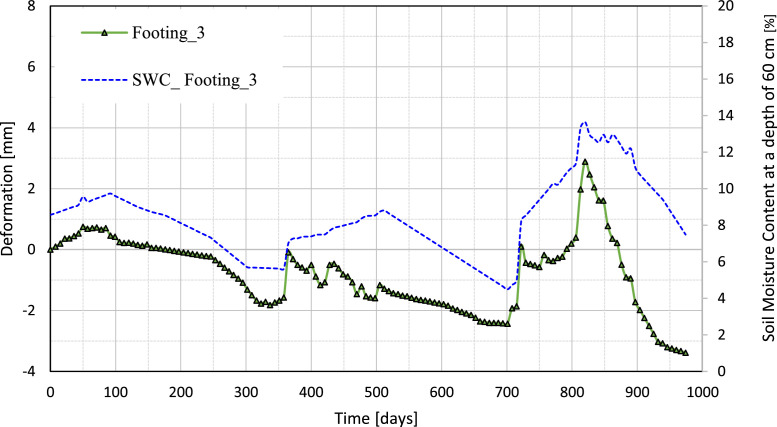
The graph shows the relation between vertical deformation and soil moisture content at a depth of 60 cm for footing_3.

**Fig. 13:**
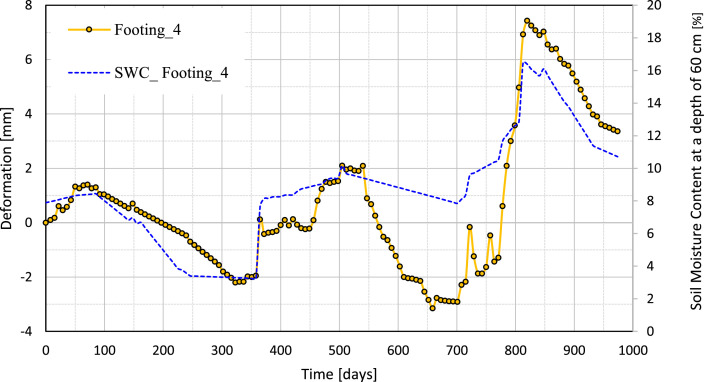
The graph shows the relation between vertical deformation and soil moisture content at a depth of 60 cm for footing_4.

Fig. 10, Fig. 11, Fig. 12, Fig. 13 illustrate the variation of the vertical deformation of footings (Footing 1, Footing _2, Footing _3, and Footing _4) plotted on the left y-axis and SWC at a depth of 60 cm plotted on the right y-axis during the whole period. These figures give a detailed comparison, showing how the variations in soil moisture at this depth correspond to the changes in vertical deformation of each footing. The curves show the influence of moisture content on the behavior of the foundation under medium-expansive soil conditions and provide insight into the interaction between soil properties and environmental factors that affect structural stability.

## Limitations

The dataset deals with real foundations located on natural medium-expansive soil, and thus the dataset applies to this type of soil. Swelling and shrinkage in expansive clay are controlled by mineralogical composition, moisture content, plasticity index, soil density, permeability, climate conditions, water table depth, overburden pressure, and chemical interaction within the soil–water system. Therefore, these parameters can be different in other locations or under other conditions, and thus the deformation behavior can also become different. Also, the obtained vertical deformation in the present dataset may differ from the results measured in laboratory tests. This is because, under natural conditions, complete saturation of the soil may not be attained due to environmental factors and dynamics of water infiltration.

## Ethics Statement

The authors have read and follow the ethical requirements for publication in Data in Brief and confirming that the current work does not involve human subjects, animal experiments, or any data collected from social media platforms.

## CRediT Author Statement

**Hawkar Hashim Ibrahim:** Conceptualization, Methodology, Validation, Investigation, Data Curation, Writing - Original Draft. **Rizgar Ali Hummadi:** Supervision, Visualization, Writing - Review & Editing.

## Data Availability

Mendeley DataField Monitoring of Foundations on Medium-Expansive Soil (Original data).
